# Embryonal Rhabdomyosarcoma of Upper Lid in 15-Year-Old Patient

**DOI:** 10.1155/2014/157053

**Published:** 2014-02-09

**Authors:** Mohammad Sharifi

**Affiliations:** Eye Research Center, Mashhad University of Medical Science, Mashhad 9195965919, Iran

## Abstract

Rhabdomyosarcoma is the most common childhood primary malignant tumor of orbit. Most of patients present between the ages of 7 and 8 years. Pure eyelid rhabdomyosarcoma is a very rare tumor with only a few reported cases in the literature. We introduce a pure embryonal rhabdomyosarcoma of upper lid in 15-year-old patient and demonstrate successful management of it.

## 1. Case Report

**Figure 1:**
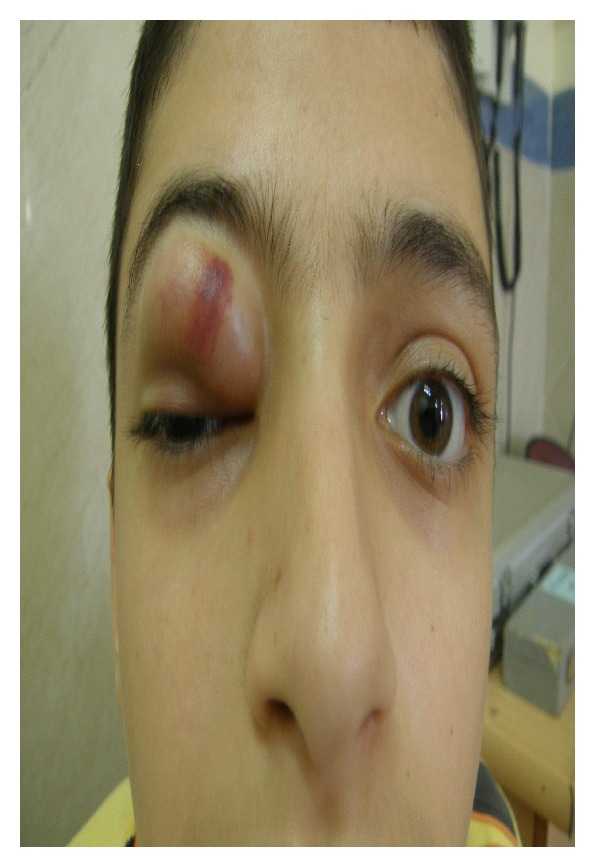
Right upper lid mass with ecchymosis led to mechanical ptosis.

A 15-year-old male patient was referred to oculoplastic service of one of teaching eye hospitals in Mashhad, Iran, due to right upper lid swelling since two months before visit. On external eye exam, the eye had nontender, nonmobile, firm, subcutaneous mass with ecchymosis of right upper lid (Figure 1). Mechanical ptosis of right upper eyelid was present with sparing of the visual axis. Eyelid margin architecture was normal. There was no history of recent trauma or family history of malignancy. Patient's right upper lid appeared normal (according to previous photograph) and swelling on right upper lid was developed 2 months before visit. The swelling increased and ecchymosis developed on lesion 1 month before visit. The lesion had been suspected of allergic reaction or insect bite and treated with topical medication but eyelid swelling did not resolve.

Other part of ophthalmic examination includes visual acuity, pupillary reflex; biomicroscopic and fundoscopic exam were normal. There was no proptosis of right eye. The left eyelids, adnexa, and globe were unremarkable. No limitation of eye movements was found.

**Figure 2:**
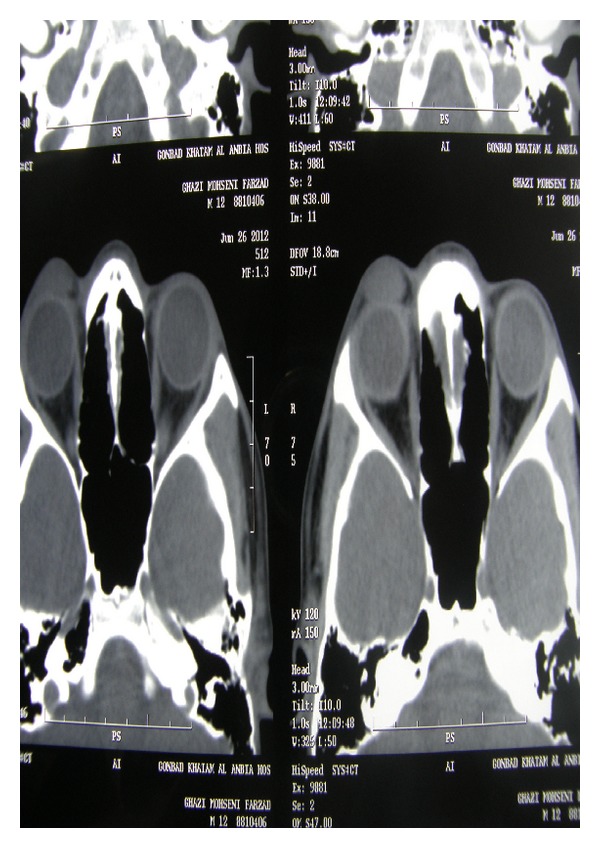
Axial and coronal view of orbital CT scan that showed well-circumscribed, uniform preseptal mass without orbital extension.

Due to chronic nature of swelling suspicious to tumoral lesion, orbital CT scan with axial and coronal view was requested, which showed well-defined, homogenous preseptal soft tissue mass without intraorbital extension or adjacent bony erosion (Figure 2).

**Figure 3:**
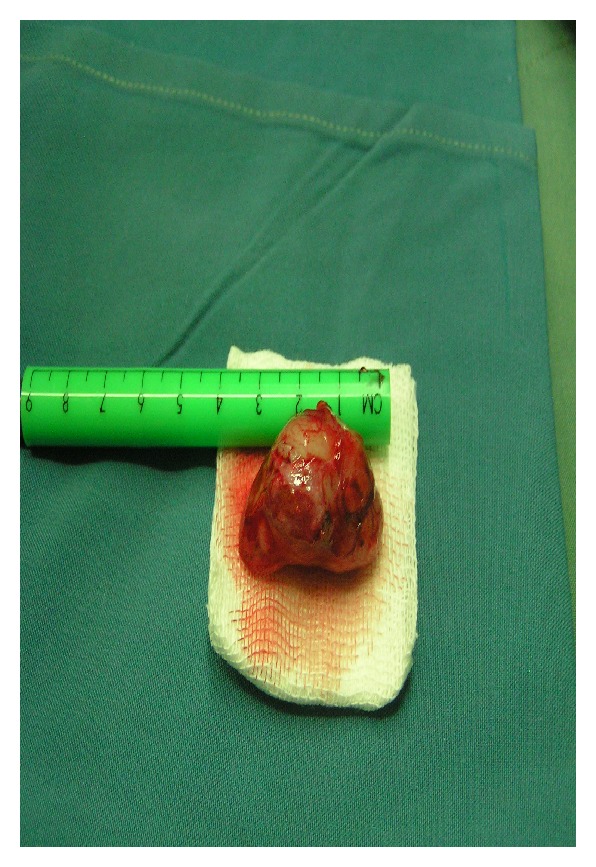
Gross view of excised tumor.

The next step was excisional biopsy of lesion. Under general anesthesia, after preparation, via lid crease incision, complete excision of mass was performed and 3 ∗ 3 ∗ 2.5 cm firm, fleshy mass was excised (Figure 3).

**Figure 4:**
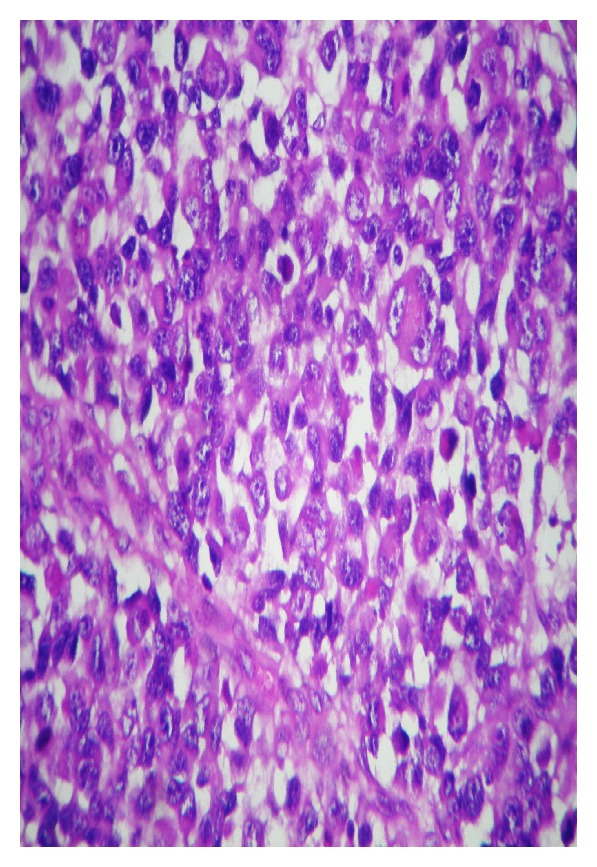
Embryonal rhabdomyosarcoma consisting almost entirely of differentiated rhabdomyoblasts (H&E staining, ×40).

**Figure 5:**
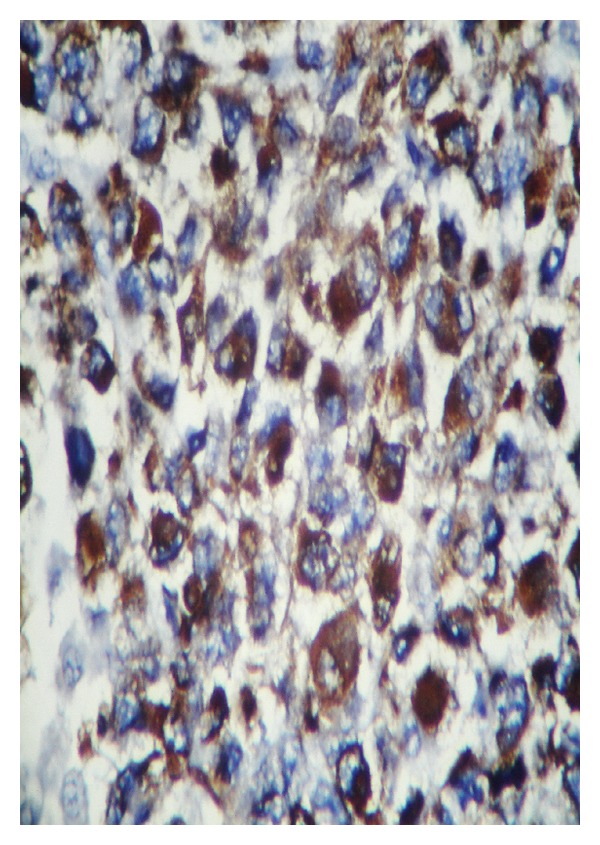
Embryonal rhabdomyosarcoma: desmin immunoreactivity in large eosinophilic cells (IHC ×40).

Histopathologic examination showed diffuse, spindle-shaped cells proliferation which were entirely rhabdomyoblasts (Figure 4). Imunohistochemical staining for desmin was highly positive (Figure 5). The diagnosis of embryonal rhabdomyosarcoma was confirmed with immunohistochemistry.

**Figure 6:**
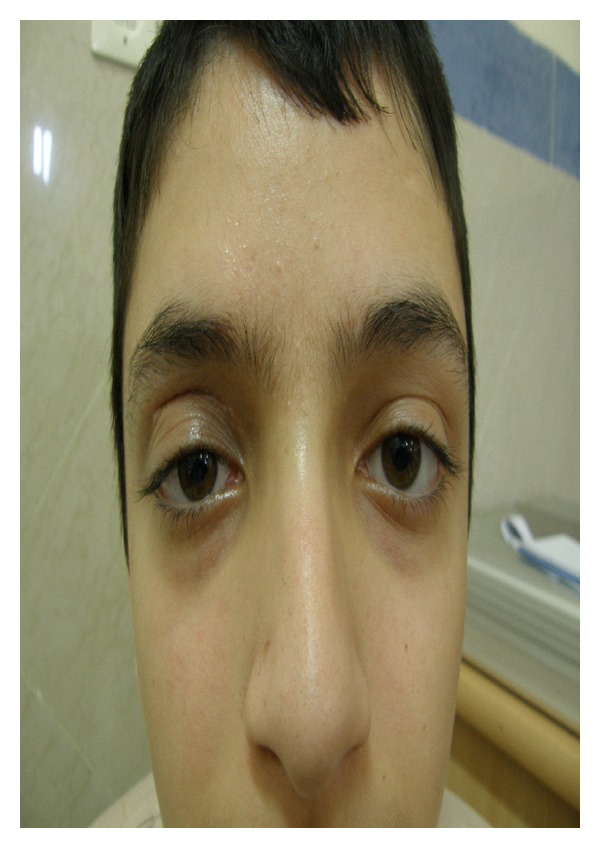
Good cosmesis and successful outcome of patient 6 months after surgery.

Patient was referred to oncologist for systemic workup. CT scans of abdomen and chest, bone scan demonstrated no evidence of metastasis or lymph node involvement. The patient underwent chemotherapy for eradication of possible residual tumor and completeness of treatment. Six months after completion of therapy, patient had good cosmetic result with no evidence of recurrence (Figure 6).

## 2. Discussion

Rhabdomyosarcoma is the most common primary orbital malignancy of childhood [1]. Classic clinical picture is sudden onset and rapid evolution of proptosis without history of previous trauma or sign of upper respiratory tract infections [2]. There is often a marked lid edema and discoloration [2, 3]. Ptosis and strabismus may also present [2, 3]. Palpable intraorbital mass may present particularly in superonasal quadrant. Tumor may rarely arise from conjunctiva term botyroid type [3]. Pure eyelid rhabdomyosarcoma, as found in our patient, is extremely rare tumor. In 2010 Jung et al. reported lower lid rhabdomyosarcoma in a 3-month-old infant which was successfully treated [4]. Bonnin et al. reported palpebral rhabdomyosarcoma of the upper eyelid in 2-year-old child treated by chemotherapy alone. Tumor regressed completely after two months and no evidence of recurrence was found at final followup [5]. Alhady et al. reported palpebral pleomorphic rhabdomyosarcoma of upper eyelid in 11-year-old teenager who presented with recurrent fungating upper eyelid mass. Excisional biopsy and adjuvant chemotherapy eradicated tumor completely [6]. Hajji et al. demonstrated embryonal rhabdomyosarcoma of upper eyelid in 7-year-old child presented with progressive enlarging mass without ecchymosis. Tumor was successfully managed by local excision and complementary chemotherapy [7]. Our case had several distinctive features including age of presentation (late onset), type of presentation (enlarging mass with ecchymosis), site of involvement (upper eyelid), and type of tumor (embryonal).

The early stages of pure eyelid rhabdomyosarcoma may be diagnosed as a benign lesion such as allergic reaction, insect bite, and hordeolum or appendage tumor. These lesions show spontaneous improvement and/or normal eyelid structure. Malignancy should be suspected whenever chronic, enlarging eyelid mass accompanied with ecchymosis is observed.

If rhabdomyosarcoma is suspected, CT and MRI can be used to define the location and extent of the tumor [3]. CT is particularly helpful if tumor has caused bony destruction, although the orbital walls remain in fact in most cases [3].

In our patient orbital CT showed uniform, localized mass in preseptal space without extension to orbit which was in favor of a benign lesion, for example, dermoid cyst or hematinic cyst.

A biopsy should be undertaken, if malignancy is suspected. It is often possible to completely remove a rhabdomyosarcoma if it had pseudocapsule [2, 3]. If this is not practical, the more effective option is the combination of surgery and chemotherapy for achieving cure [1, 3].

Immunohistochemical staining helped to confirm the diagnosis. The most useful immunohistochemical marker includes antibodies against muscle-specific actin and desmin, which show the greatest specifity, vimentin shows a positive reaction in most rhabdomyosarcoma but is less specific for this tumor because it can be positive in other malignancy. Other reports have described presentation between range of 3 weeks to 78 years old and retain positive reaction even in poorly differentiated rhabdomyoblasts [4]. Vimentin shows a positive reaction in most cases but it is less specific and can be positive in a variety of other neoplasms [8]. Cross-striations are often not visible on light microscopy and may be more readily apparent on electron microscopy [3]. Unusual aspects of this case were the patient's age at presentation and pure upper lid involvement. Ocular rhabdomyosarcoma is primarily of a disease of young children with mean age 7-8 years old [3]. Other reports have described presentation between range of 3 weeks to 78 years old. Pleas correct in text [9, 10].

Before 1926 the standard treatment of orbital rhabdomyosarcoma was orbital exenteration and the survival rate was poor [3]. Since 1965, radiation therapy and systemic chemotherapy have become the main stays of primary treatment, based on the guidelines set forth by intergroup rhabdomyosarcoma studies [3].

These studies have unusually been directed to rhabdomyosarcoma from all locations. Metastasis from orbital rhabdomyosarcoma is relatively uncommon and regional lymph node metastasis is also rare because orbit is largely void of lymphatic [4]. However, compared with orbital rhabdomyosarcoma, tumor located in the conjunctiva or eyelid can go in access to lymphatic channels and easily metastasize to preauricular or cervical lymph nodes [3].

Although the orbit is the most common site of ocular rhabdomyosarcoma and mean age at diagnostics is about 7-8 years old, we report an upper eyelid embryonal rhabdomyosarcoma in 15-year-old man presented as enlarging right upper lid mass accompanied by ecchymosis similar to benign lesion such as subcutaneous hemorrhage. Therefore, ophthalmologists must be suspicious about rhabdomyosarcoma in any patient with eyelid swelling or mass and ecchymosis without history of trauma.
